# Counting Replication Origins to Measure Growth of Pathogens

**DOI:** 10.3390/antibiotics9050239

**Published:** 2020-05-08

**Authors:** Godefroid Charbon, Maria Schei Haugan, Niels Frimodt-Møller, Anders Løbner-Olesen

**Affiliations:** 1 Department of Biology, University of Copenhagen, Ole Maaløes vej 5, 2200 Copenhagen N, Denmark; godefroid.charbon@bio.ku.dk; 2 Department of Medical Microbiology, St. Olavs Hospital, Trondheim University Hospital, 7006 Trondheim, Norway; maria.schei.haugan@stolav.no; 3 Department of Clinical Microbiology, Rigshospitalet, 2100 Copenhagen, Denmark; niels.frimodt-moeller@regionh.dk

**Keywords:** growth rate, pathogen, in situ, antibiotics, DNA

## Abstract

For the past several decades, the success of bacterial strains in infecting their host has been essentially ascribed to the presence of canonical virulence genes. While it is unclear how much growth rate impacts the outcome of an infection, it is long known that the efficacy of the most commonly used antibiotics is correlated to growth. This applies especially to β-lactams, whose efficacy is nearly abolished when cells grow very slowly. It is therefore reasonable to assume that a niche or genetic dependent change in growth rate could contribute to the variability in the outcome of antibiotic therapy. However, little is known about the growth rate of pathogens or their pathotypes in their host.

## 1. Introduction

**Figure 1:**
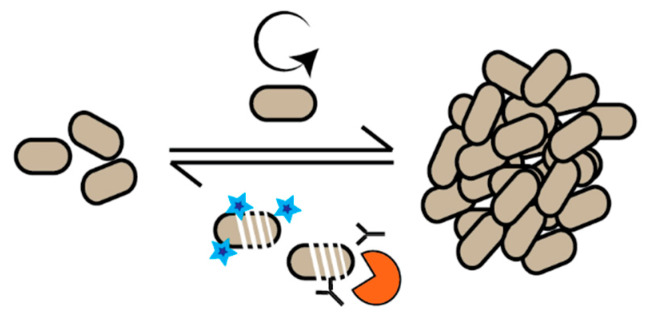
In situ population dynamics. The size of a bacterial population is determined by how fast individual cells grow and how fast the cells are killed by antibiotics or immune system defense mechanisms.

Recently, efforts have been made to understand the growth dynamics of bacteria in situ. Classically, bacterial population dynamics are observed by viable cell counting using colony forming units (CFUs). This gives a good indication of the size of the bacterial population at the time of sampling. Although, because a change in bacterial load results from both cell growth and cell death, it fails to measure how fast bacteria are growing (Figure 1).

Simply put, the same CFU count can be obtained from a population of cells growing fast with a high death rate, as from a population of cells growing slowly with a low death rate. In test tube experiments, growth is usually measured by change in optical density. This is obviously not feasible in in-host setups, and death rate attributed to immune system response or antibiotics is not taken into account.

## 2. DNA Replication as a Growth Marker

**Figure 2:**
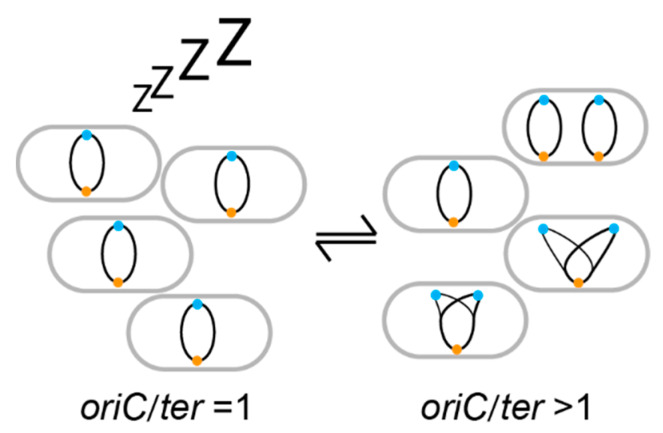
Origin of replication/terminus (*oriC*/*ter*) ratio as a measure of growth rate. Non-growing cells (left) possess fully replicated chromosomes with one *oriC* (blue ball) and one *ter* (orange ball). During the DNA duplication period of growing cells (right), there are more *oriC* than *ter*. Thus, the *oriC*/*ter* ratio of the population is above 1.

Lately, several methods have been applied to infer the in situ growth rate of bacteria [1,2,3,4,5,6,7,8]. One of these methods takes advantage of the coupling of DNA replication with growth rate. In *Escherichia coli* and other bacteria, initiation of DNA replication is linked to growth rate changed by nutrient availability [9,10]. In *E. coli*, it takes a minimum of ~60 min to finish a cell cycle. The bidirectional DNA duplication starts from the origin of replication (*oriC*) and finishes at the terminus (*ter*), a minimum of 40 min later (C period). Subsequently, it takes ~20 min for the cell to divide (D period). Thus, the *oriC* copy number is increased for a certain amount of time before the *ter* copy number increases. In cells growing very slowly, DNA is replicated in a small proportion of the cell cycle, while during fast growth, cells are born with overlapping rounds of DNA replication. The upshot is that the ratio of *oriC* to *ter* reflects the growth rate of a bacterium. Invariably, in a population of actively replicating cells, *oriC*/*ter* is above 1 (Figure 2).

Already, this information becomes valuable to distinguish growing cells from non-growing cells; non-growing cells do not replicate their DNA, thus *oriC*/*ter* is equal to one. Cells growing faster than it takes to finish a cell cycle (C+D) are born with chromosomes in the process of being replicated, thus multiple *oriC* per one *ter*. For example, a population of *E. coli* growing in rich media or in situ can have eight *oriC* in a single cell and an *oriC*/*ter* ratio of about four. The faster the cells grow the more *oriC* per *ter*. This remarkable correlation has long been acknowledged, but only recently has it been used as a proxy to measure growth of pathogens in their host. This has been done by quantitative PCR amplification of *oriC* and *ter* [11,12,13], marker frequency analysis by whole genome sequencing [3,4,14,15,16], or direct microscopic visualization of fluorescently labeled *oriC* and *ter* in live cells [11,13], all of which will be referred to as marker frequency analysis (MFA) in the text. The value of the information obtained from such techniques is made apparent in some examples from recent studies that will be discussed below.

## 3. Dynamics in Specific Niches

The first example of this application comes from the study of uropathogenic *E. coli* (UPEC) causing urinary tract infections. Human urine was long thought to be too toxic to accommodate any kind of growth. Yet, growth of UPEC has been measured to be extremely fast (faster or similar to growth in Lysogeny broth rich medium) [4]. This shows that the bacterium not only thrives in the urinary tract, but also implies that fast growth could be mechanistically required for bacterial maintenance in niches with fast fluidic discharge. Using MFA for measuring growth, a similar mechanism was proposed for *Staphylococcus aureus* growing in the nasal cavity [2]. There are also a significant number of bacteria that cannot be grown in synthetic media, their existence only revealed by DNA sequencing. Here, growth rate derived from MFA has been valuable to obtain very basic data on their growth in situ [14].

## 4. Dynamics During an Infection Process

In the second example, focus is placed on the population dynamics during infection: Are there few bacteria at the infection site because they grow slowly or is it because they are cleared by the immune system despite fast growth? (Figure 1). Common to the few studies made so far, is that there is a not a clear correlation between the bacterial load (CFU per gram) and the growth rate measured at the site of infection. This is exemplified in an analysis of the gut microbiome in an infant developing necrotizing enterocolitis, revealing that the load of *Clostridium* species was very low despite rapid bacterial growth [14]. In another example following the dynamics of a mouse peritoneal infection over time, the load of *E. coli* at the primary site of infection increased and reached a plateau [11]. This apparent plateau in bacterial load is made up of both slowly growing and non-growing cells kept in check by the host immune system. A low load of *E. coli* was also found in the bloodstream; however, the growth rate of the bacteria in the blood mirrored the one found in the peritoneal cavity throughout the infection. This supports the concept that the bacteria found in the bloodstream are not establishing themselves but represent a mere spill-over from the site of infection. The conclusion from this study was that the mouse immune system is presumably capable of better clearance of *E. coli* in the bloodstream than in the peritoneum.

## 5. Dynamics During Antibiotic Therapy

**Figure 3:**
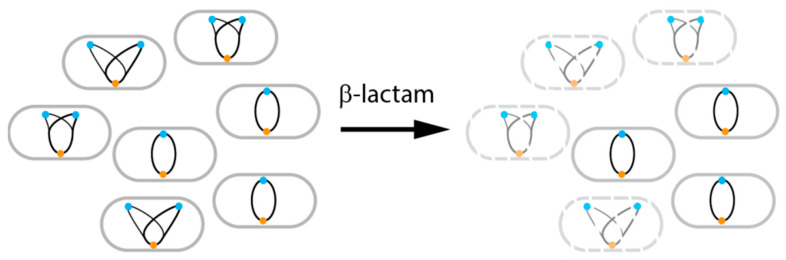
Measure of growth rate as read out for antibiotic efficacy. Selective action of β-lactams on actively growing cells (more than one *oriC* per *ter*) is represented.

Finally, knowledge about in situ bacterial growth could be used to select appropriate antibiotics or predict their efficacy. For example, this is seen when analyzing the efficacy of antibiotics in eradicating an *E. coli* infection from the mouse peritoneum [13]. Before antibiotic treatment, the bacterial population was made up of non-growing and slowly growing cells. Treatment with ceftriaxone, a β-lactam drug, preferentially eliminated growing cells, consistent with what is known about this class of antibiotics [17,18] (Figure 3). This type of analysis is now expanded to analyze *E. coli* during human urinary tract infection therapy [12].

## 6. Concluding Remarks

While it is established that a variety of *E. coli* can cause human infection, it is now also clear that the pathogens do not invariably grow at the same pace. The use of MFA to measure bacterial growth dynamics during infection provides a potential for future patient-bacterium specific antibiotic treatment regimens.
